# Effect of Coenzyme Q10 Supplementation on Cardiac Function and Quality of Life in Patients with Heart Failure: A Randomized Controlled Trial

**DOI:** 10.3390/jcm14113675

**Published:** 2025-05-23

**Authors:** Olivia Bodea, Eugen Radu Boia, Laura Maria Craciun, Mihaela Daniela Valcovici, Alexandru Catalin Motofelea, Andreea Mara Munteanu, Caius Glad Streian, Gheorghe Nicusor Pop, Simona Ruxanda Dragan

**Affiliations:** 1 Department of Doctoral Studies, “Victor Babes” University of Medicine and Pharmacy Timisoara, Eftimie Murgu Square No. 2, 300041 Timisoara, Romania; olivia-maria.bodea@umft.ro; 2 Discipline of Internal Medicine and Ambulatory Care, Prevention and Cardiovascular Recovery, Department VI-Cardiology, “Victor Babes” University of Medicine and Pharmacy, 3000041 Timisoara, Romania; laura.craciun@umft.ro (L.M.C.); simona.dragan@umft.ro (S.R.D.); 3 Department of Ear, Nose and Throat, Faculty of Medicine, “Victor Babeș” University of Medicine and Pharmacy Timisoara, 2 Eftimie Murgu Sq., 300041 Timisoara, Romania; 4 Department of Cardiology, “Victor Babes” University of Medicine and Pharmacy, 300041 Timisoara, Romania; mihaeladanielacardio@gmail.com (M.D.V.); streian.caius@umft.ro (C.G.S.); 5 Center for Molecular Research in Nephrology and Vascular Disease, Faculty of Medicine, “Victor Babes” University of Medicine and Pharmacy, 300041 Timisoara, Romania; alexandru.motofelea@umft.ro; 6 Department V, Internal Medicine I—Discipline of Internal Medicine IV, Center of Advanced Research in Cardiology and Hemostasology, “Victor Babes” University of Medicine and Pharmacy, Eftimie Murgu Sq. No. 2, 300041 Timisoara, Romania; munteanu.andreea@umft.ro; 7 Center for Modeling Biological Systems and Data Analysis (CMSBAD), “Victor Babes” University of Medicine and Pharmacy, 300041 Timisoara, Romania; pop.nicusor@umft.ro

**Keywords:** heart failure, coenzyme Q10 supplementation, MLHFQ, quality of life, global longitudinal strain

## Abstract

**Background/Objectives:** Heart failure remains a complex syndrome with high morbidity and mortality, highlighting the urgent need for alternative treatments that address underlying bioenergetic impairments. CoQ10, which plays a crucial role in mitochondrial ATP production, has shown promising results in small studies, although larger trials are needed to confirm its efficacy. **Results:** This randomized controlled trial investigated the effects of coenzyme Q10 (CoQ10) supplementation on cardiac function and quality of life in heart failure patients. A total of 120 patients were randomly assigned to receive either CoQ10 (2 × 60 mg daily) or a placebo for six months. Baseline characteristics were similar between groups. The primary outcomes were changes in global longitudinal strain (GLS) and left ventricular ejection fractions (LVEFs), while secondary outcomes included improvements in functional capacity and quality of life. At the 6-month endpoint, the CoQ10 group showed significant improvements in GLS (−11.7% to −14.9%, *p* < 0.001), NT-proBNP levels (815.6 vs. 1378.5 pg/mL, *p* = 0.012), blood pressure, and 6 min walk test distance (349.3 vs. 267.0 m, *p* = 0.008) compared to the placebo group. LVEFs improved slightly in the CoQ10 group (38.9% to 40.6%, *p* = 0.170) but remained unchanged in the placebo group. **Conclusions:** These findings suggest that CoQ10 supplementation may improve cardiac function, reduce cardiac stress, and enhance functional capacity and quality of life in heart failure patients. Further research is needed to optimize dosage and identify the subgroups that may benefit most from CoQ10 therapy.

## 1. Introduction

Despite significant advancements in medical and device-based therapies, heart failure (HF) remains a complex, multifaceted syndrome that continues to affect millions globally, with high morbidity and mortality rates [1,2,3]. The chronic nature of HF and its poor outcomes highlight the need for alternative therapies that address underlying bioenergetic impairments, which are often inadequately targeted by current treatment approaches.

CoQ10 plays a critical role in the mitochondrial respiratory chain, where it facilitates ATP production. However, despite the promising results from small-scale research, few trials have had sufficient statistical power to demonstrate significant improvements in heart failure outcomes [4,5,6,7,8].

Mitochondrial dysfunction is a key feature of chronic HF, leading to a maladaptive bioenergetic response and a progressive reduction in energy reserves, irrespective of the underlying cause [5]. Ubiquinone, or CoQ10, is a key component of this process, and its role in cardiac bioenergetics is well established [6]. Found naturally in most aerobic organisms, including humans, CoQ10 is essential for maintaining mitochondrial function. By 1974, the Japanese government had approved CoQ10 for treating congestive heart failure, further demonstrating its therapeutic potential [9].

Since the late 1980s, randomized controlled trials (RCTs) investigating CoQ10 in heart failure patients have been conducted. While these trials have shown promise, many have been too underpowered to significantly address clinical outcomes, leaving gaps in the evidence [10]. The potential for CoQ10 to improve outcomes in HF patients is particularly compelling given its well-documented role not only in cardiovascular therapy but also in complementary and alternative medicine for conditions such as cancer, periodontal disease, and others believed to benefit from antioxidant supplementation [11].

CoQ10 is crucial for ATP production and cellular energy mechanisms as part of the mitochondrial respiratory chain. Additionally, it is one of two naturally occurring antioxidants in low-density lipoproteins (LDLs), with vitamin E being the other [6,12]. Researchers have demonstrated that CoQ10 supplementation enhances cardiac efficiency in patients with coronary artery disease, as evidenced by an increase in oxygen consumption per unit of ATP produced [13].

The rationale for utilizing CoQ10 in HF patients stems from its ability to address the energy depletion seen in failing hearts. Low endogenous CoQ10 levels have been directly linked to decreased contractile function in patients with HF. Endomyocardial biopsy samples from HF patients, collected in accordance with the New York Heart Association (NYHA) guidelines, have revealed deficiencies in CoQ10 levels in both plasma and myocardial tissue. These findings indicate a direct correlation between disease severity and myocardial CoQ10 deficiency. Importantly, oral administration of 90 mg of CoQ10 has been shown to reduce the severity of HF symptoms [14,15].

Thus, given the established link between mitochondrial dysfunction and heart failure progression, as well as the observed deficiency of CoQ10 in HF patients, this study aims to evaluate the impact of CoQ10 supplementation on key clinical parameters, including NT-proBNP levels, ejection fractions, and functional capacity. We hypothesized that CoQ10 supplementation would significantly improve these outcomes compared to a placebo, providing further evidence for its use as a therapeutic intervention in HF management.

## 2. Materials and Methods

### 2.1. Data Collection

**Figure 1:**
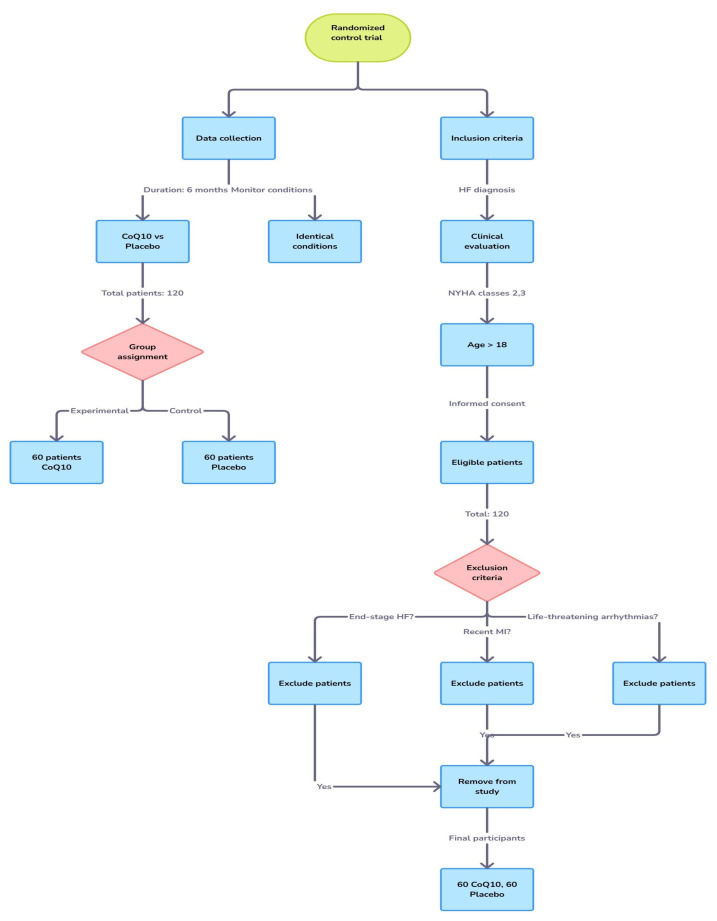
Flowchart of this study.

This prospective study was performed over a period of six months and aimed at comparing the effects of a CoQ10 supplement versus a placebo in patients diagnosed with heart failure (HF). The trial included a total of 120 patients, randomly assigned to two groups: 60 patients were in the experimental group receiving CoQ10 supplementation and 60 patients were in the control group receiving a placebo. This study followed a parallel design, with both groups monitored under identical conditions (Figure 1).

### 2.2. Inclusion and Exclusion Criteria

The inclusion criteria required patients to be diagnosed with heart failure based on clinical evaluation and imaging studies, and to be categorized under NYHA classes II and III. Patients had to be older than 18 years and able to give informed consent. Patients with end-stage heart failure, a recent myocardial infarction (within the past 6 months), or life-threatening arrhythmias were excluded from this study. A total of 120 eligible patients (50% men, with a mean age of 67.09 ± 13.53 years) were enrolled, and they were evenly randomized into the Q10 or placebo group.

### 2.3. Randomization and Blinding

Patients were randomly assigned to either the CoQ10 or placebo group using a computer-generated randomization sequence. Both the patients and investigators were blinded to the treatment assignments throughout the study to avoid bias. The placebo and CoQ10 capsules were identical in appearance, and neither the patients nor the clinical staff knew which treatment was being administered.

Baseline Characteristics:

Baseline characteristics, including demographic, clinical, and laboratory data, were collected at the start of this study for both groups. The variables assessed included age, BMI, smoking status, NYHA class on admission, and comorbidities such as diabetes mellitus type 2 atrial fibrillation (FiA), and chronic obstructive pulmonary disease (COPD). Cardiac function was evaluated by echocardiography, and relevant parameters such as left ventricular ejection fraction (FE), end-diastolic volume (VTD), end-systolic volume (VTS), and GLS (global longitudinal strain) were measured. Respiratory and metabolic variables, including NT-proBNP, hemoglobin (Hb), and renal function (RFG and creatinine), were also documented.

Intervention:

Patients in the experimental group received a daily dose of CoQ10 (2 × 60 mg), while the control group received a placebo of identical appearance. Treatment was administered for the duration of the six-month study. Both groups continued their standard heart failure treatments, including beta-blockers (BBs), angiotensin-converting enzyme inhibitors (IECA), angiotensin II receptor blockers (BRA), aldosterone antagonists (AAs), and SGLT inhibitors. Any changes in medication were documented at discharge.

Outcome Measures:

The primary outcome measures included changes in global longitudinal strain (GLS) and left ventricular ejection fraction (LVEF) to assess cardiac function. Secondary outcomes encompassed improvements in functional capacity and quality of life, as evaluated by the Minnesota Living with Heart Failure Questionnaire (MLHFQ). Secondary outcomes also included the number of heart-failure rehospitalizations over six months. In this period, 2 patients in the CoQ10 arm and 3 in the placebo arm were rehospitalized.

### 2.4. Statistical Analysis

Continuous variables, such as age, BMI, NT-proBNP level, and GLS, were summarized as means with standard deviations (SDs). Categorical variables, including NYHA class and the presence of comorbidities, were presented as frequencies and percentages. Changes in NT-proBNP levels, GLS, ejection fractions (EFs), and functional capacity (6MWT) were assessed using paired t-tests to compare the baseline and endpoint measurements within each group. Between-group comparisons were conducted using independent sample t-tests for continuous outcomes and Chi-square tests for categorical outcomes. Parameters such as oxygen saturation, BMI, and Minnesota Living with Heart Failure Questionnaire (MLHFQ) scores were analyzed using paired and independent t-tests for within-group and between-group comparisons, respectively. Subgroup analyses were performed to evaluate the differential effects of CoQ10 based on comorbidities (diabetes, atrial fibrillation) and NYHA classification at baseline.

A power sample size analysis was conducted a priori to ensure this study had at least 80% power to detect statistically significant differences.

Missing values were addressed using multiple imputation techniques to ensure robustness and minimize bias in the analysis. The sensitivity analyses compared the results from complete-case analyses with those from the imputed datasets.

A *p*-value < 0.05 was considered statistically significant for all analyses. Confidence intervals (CIs) at the 95% level were reported alongside point estimates to provide measures of precision.

To assess the reliability of speckle-tracking echocardiography measurements over time, we evaluated both inter- and intra-observer variability. Fifteen echocardiographic studies were randomly selected and re-analyzed: intra-observer variability was determined by having the same investigator repeat measurements after a two-week interval, and inter-observer variability was determined by an independent second investigator. Intraclass correlation coefficients (ICCs) were calculated for GLS, EF, VTD, and VTS measurements. To further guard against potential errors, a cross-check was performed with both investigators on this subset of 15 cases. This process demonstrated excellent reproducibility (ICC > 0.85 for all parameters) and supports the robustness of our echocardiographic findings.

All statistical analyses were conducted using R (version 4.2.0 R Foundation for Statistical Computing, Vienna, Austria).

This methodology provided a rigorous framework to assess the efficacy and safety of CoQ10 supplementation in improving outcomes for heart failure patients while minimizing bias and ensuring statistical integrity.

### 2.5. Ethical Considerations

This study was conducted in accordance with the Declaration of Helsinki, and ethical approval was obtained from the institutional review board (approval number 2099/16 March 2022). Written informed consent was obtained from all patients before their participation in this study.

## 3. Results

A total of 120 patients were enrolled in this study, with 60 participants assigned to the CoQ10 group and 60 to the placebo group for comparison. The average age was 66.5 years in the CoQ10 group and 67.7 years in the placebo group, with no statistically significant difference (*p* = 0.624). Baseline characteristics, including environment, height, weight, BMI, and smoking status, were similar between the two groups, ensuring balanced conditions for treatment. Among the study population, 52.1% of patients were classified as NYHA class II, and 47.9% as NYHA class III. The NYHA class distribution was consistent between groups (*p* = 0.786).

The prevalence of Type 2 diabetes was also similar between the CoQ10 group (41.7%) and the placebo group (45.0%), with no significant difference (*p* = 0.713). Other comorbidities, such as atrial fibrillation (CoQ10: 55.0%, Placebo: 40.0%, *p* = 0.100) and COPD (CoQ10: 36.7%, Placebo: 23.3%, *p* = 0.111) showed no significant differences between the groups.

The functional capacity of patients, as assessed by the 6MWT, showed no significant difference between groups. The CoQ10 group walked an average distance of 299.4 m compared to 292.1 m in the placebo group (*p* = 0.49). Laboratory data revealed no significant difference NT-proBNP levels, with similar values in the CoQ10 group (1397.4 pg/mL) and the placebo group (1401.9 pg/mL, *p* = 0.987). The MLHFQ scores (57.6, 54.0, 55.8; *p* = 0.227) indicate a similar quality of life across groups, with no statistically significant differences (Table 1).

### 3.1. Comparison of Medication Usage at Admission–Discarge

Class-specific guideline-directed medical therapy (GDMT) compliance—defined as an uninterrupted prescription of each drug class from discharge through six months—was high. Specifically, β-blockers were maintained in 83% of CoQ10 patients versus 78% of those receiving a placebo; RAAS blockers were maintained in 28% of CoQ10 patients versus 25% of placebo patients. MRAs were maintained in 42% of CoQ10 patients versus 31% of placebo patients; SGLT2 inhibitors were maintained in 23% of CoQ10 patients versus 28% of placebo patients. And ARBs were maintained in 12% of CoQ10 patients versus 17% of placebo patients.

**Figure 2:**
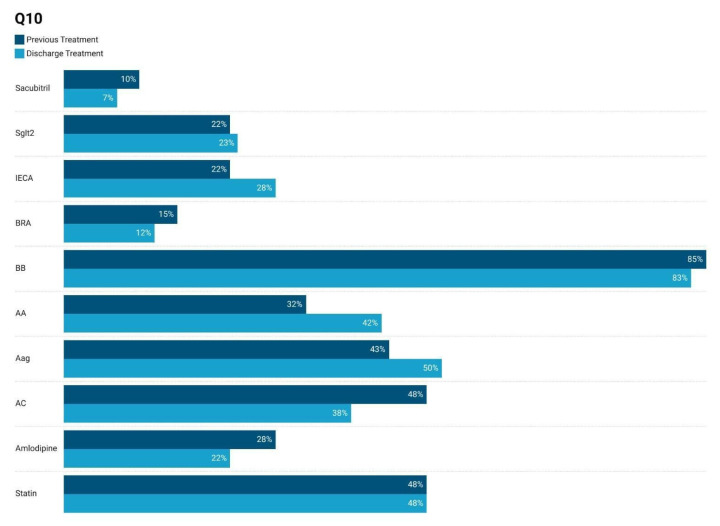
Comparison of medication usage before and after discharge in heart failure patients receiving CoQ10 supplementation at 6 months.

Figure 2 illustrates medication usage before and after discharge in heart failure patients receiving CoQ10. There was an increase in the use of ACE inhibitors (22% to 28%), aldosterone antagonists (32% to 42%), and anticoagulants (38% to 48%) post-discharge, reflecting optimized heart failure therapy. Beta-blocker use remained consistently high (85% to 83%), while ARB usage decreased slightly (15% to 12%). Statin usage remained stable at 48%. These changes align with evidence-based heart failure management guidelines.

**Figure 3:**
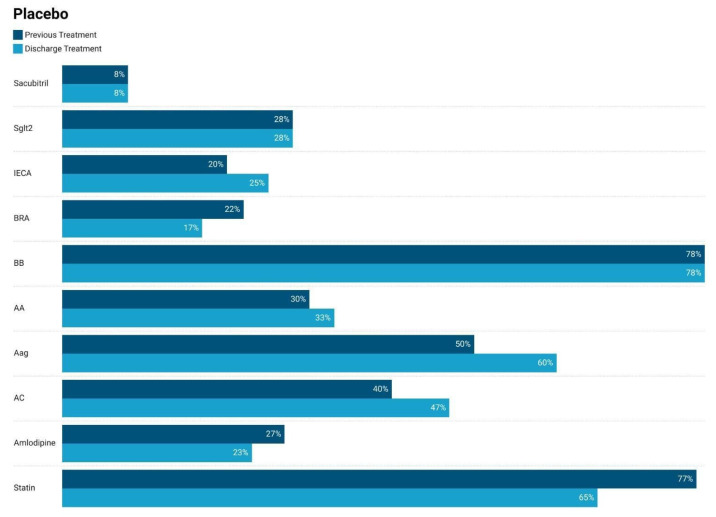
Comparison of medication usage before and after discharge in heart failure patients receiving placebo supplementation at 6 months.

Figure 3 presents a similar analysis for the placebo group. Medication use changes were minimal. ACE inhibitor use increased from 20% to 25%, aldosterone antagonist use increased slightly from 30% to 31%, and anticoagulant use increased from 40% to 47%. Statin usage rose from 65% to 77%, while beta-blocker usage remained constant at 78%. The slight variations reflect routine management adjustments.

Figure 3 compares medication use before and after discharge in the heart failure patients receiving a placebo. There are minor changes in medication usage post-discharge. Sacubitril and SGLT2 inhibitor use remained consistent at 8% and 28%, respectively. ACE inhibitor use increased slightly from 20% to 25%, while ARB use decreased from 22% to 17%. Beta-blocker use remained steady at 78%. Aldosterone antagonists showed a slight increase from 30% to 31%, while angiotensin receptor neprilysin inhibitor use increased from 50% to 60%. Anticoagulant use also increased from 40% to 47%. Statins showed a slight rise from 65% to 77%. These results suggest minimal adjustments in therapy while maintaining adherence to guideline-recommended treatments.

Patients who were included in this study kept the medication recommended at discharge.

### 3.2. Endpoint Results (6-Month Evaluation)

Oxygen saturation levels remained stable across both groups. The initial SaO2 level was 98.6 ± 0.6% in the CoQ10 group and 96.5 ± 4.1% in the placebo group (*p* = 0.964). The final SaO2 level was also similar, with values of 98.4 ± 0.7% for the CoQ10 group and 95.2 ± 4.6% for the placebo group (*p* = 0.936). Echocardiographic findings revealed that Global Longitudinal Strain (GLS) improved significantly in the CoQ10 group, from −11.7 ± 4.4% at baseline to −14.9 ± 3.7% at the endpoint (*p* < 0.001), while the placebo group showed minimal improvement (−11.2 ± 3.8% to −11.6 ± 3.8%, *p* = 0.497). Left Ventricular Ejection Fractions (LVEFs) improved slightly but not significantly in the CoQ10 group (38.9 ± 6.9% to 40.6 ± 6.6%, *p* = 0.170) and remained unchanged in the placebo group (38.4 ± 7.2% at both time points, *p* = 0.960).

NT-proBNP levels, a key marker of heart failure severity, decreased significantly in the CoQ10 group (815.6 ± 957.4 pg/mL) compared to the placebo group (1378.5 ± 1492.4 pg/mL, *p* = 0.012), suggesting reduced cardiac stress in the CoQ10 group.

Systolic Blood Pressure (SBP) was significantly lower in the CoQ10 group at the endpoint (124.2 ± 13.3 mmHg) compared to the placebo group (134.9 ± 17.8 mmHg, *p* < 0.001). Similarly, Diastolic Blood Pressure (DBP) was lower in the CoQ10 group (74.2 ± 9.2 mmHg) than in the placebo group (79.9 ± 10.2 mmHg, *p* < 0.001). Functional capacity, as assessed by the 6MWT, showed a significant improvement in the CoQ10 group, with patients covering an average of 349.3 ± 100.6 m compared to 267.0 ± 96.5 m in the placebo group (*p* = 0.008). This improvement of 82.3 m is clinically meaningful for heart failure patients.

At the 6-month endpoint, several significant differences between the CoQ10 and placebo groups were observed in terms of the hemodynamic, metabolic, and functional parameters. Potassium (K) and sodium (Na) concentrations were not significantly different between groups. Potassium levels were 4.0 ± 0.6 mmol/L in the CoQ10 group and 4.4 ± 0.6 mmol/L in the placebo group (*p* = 0.722), while sodium levels were 140.6 ± 4.1 mmol/L in the CoQ10 group and 141.0 ± 2.9 mmol/L in the placebo group (*p* = 0.295). Renal function, as measured by eGFR, was significantly better in the CoQ10 group (73.1 ± 16.7 mL/min) compared to the placebo group (62.1 ± 24.2 mL/min, *p* = 0.014).

Medication usage showed some differences between groups. At baseline, 77% of the placebo group and 48% of the CoQ10 group were taking statins (*p* = 0.001). At discharge, statin use remained higher in the placebo group (65%) compared to the CoQ10 group (48%), though the difference was not statistically significant (*p* = 0.065).

CoQ10 supplementation led to significant improvements in renal function, NT-proBNP levels, blood pressure, GLS, and functional capacity, along with clinically meaningful reductions in cardiac stress. These results demonstrate the potential benefits of CoQ10 in improving outcomes for heart failure patients over a 6-month period. Adjustments to medication use, particularly the use of statins, reflected consistent patterns in routine management but did not significantly impact these outcomes (Table 2 and Table 3).

**Figure 4:**
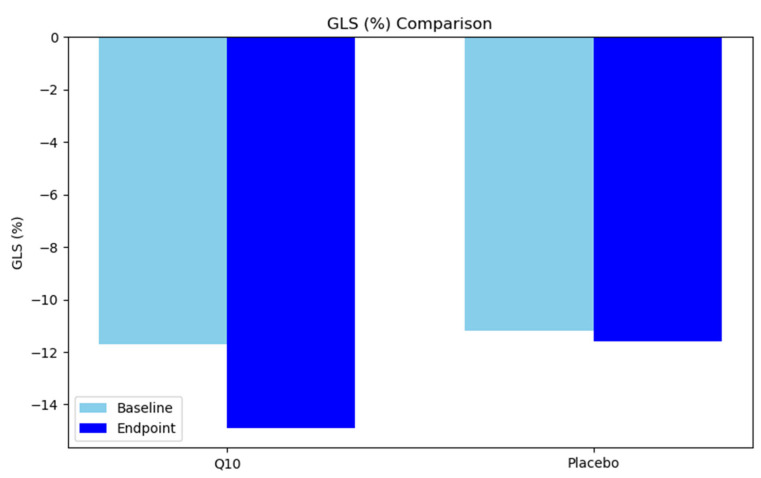
Comparison of GLS values at baseline and post-treatment.

The bar chart illustrates the mean Global Longitudinal Strain (GLS) values for both the CoQ10 and placebo groups before (lighter bars) and after (darker bars) the intervention. An improvement in GLS is evident, with the CoQ10 group showing a more pronounced increase compared to the placebo group, suggesting a beneficial effect of supplementation on myocardial function (Figure 4).

## 4. Discussion

This study demonstrated that CoQ10 supplementation led to significant improvements in several clinical and functional parameters, including a reduction in NT-proBNP levels, improved GLS, increased EF, and better performance scores for the 6MWT and MLHFQ (Minnesota Living with Heart Failure Questionnaire) compared to a placebo group. These results suggest that CoQ10 may play a beneficial role in improving both cardiac function and quality of life in patients with heart failure.

The results of this study align with previous research, particularly the findings from the Q-SYMBIO trial by Mortensen et al. [10] which showed significant improvements in functional class, NT-proBNP levels, and major adverse cardiovascular events (MACEs) after two years of Q10 supplementation. Mortensen et al. also found reduced mortality rates and improved exercise tolerance, supporting the results of this study, where GLS, ejection fraction, and 6MWT distance improved with Q10 supplementation [10]. Additional trials have also demonstrated similar benefits, including a study involving 115 high-risk congestive heart failure patients. In this trial, CoQ10 treatment led to symptom improvement across all patients, a reduction in blood pressure for 80% of participants, and improved diastolic function [8]. The consistency of these findings strengthens the case for CoQ10 as a viable therapeutic option in heart failure management.

In contrast, Watson et al. [16] found no significant differences in MLHFQ scores after three months of CoQ10 supplementation. The Japanese trial reported that 53% of patients became asymptomatic after four weeks, further supporting CoQ10’s potential to alleviate symptoms in a dose-dependent manner [17,18]. In our study, CoQ10 was administered at 60 mg/day.

The improvements observed in this study can be attributed to the role of CoQ10 as an essential cofactor in mitochondrial energy production and its potent antioxidant properties, which help reduce oxidative stress in heart failure patients. By improving myocardial energy metabolism, CoQ10 supplementation likely contributes to enhanced left ventricular function, as seen in the increase in EF and GLS improvements. These benefits are also supported by the findings from a large-scale Italian multicenter trial, where 2664 patients with NYHA functional class II or III heart failure experienced improvements in a range of symptoms, including cyanosis, edema, dyspnea, and palpitations, following three months of CoQ10 supplementation [19]. The reduction in NT-proBNP levels further supports this, as lower levels indicate reduced ventricular strain and improved cardiac health.

The findings of this study hold significant clinical relevance. The observed improvements in GLS (−11.72% from to −14.91% *p* < 0.001) and EF (from 38.87% to 40.57%, *p* < 0.001) suggest enhanced left ventricular function, which is a crucial predictor of HF progression and outcomes. Moreover, the 50 m increase in the 6MWT (*p* < 0.001) reflects improved functional capacity, a key outcome in HF management. The MLHFQ score reduction by seven points (*p* < 0.001) further emphasizes an enhancement in patients’ quality of life. These findings suggest that CoQ10 may serve as a beneficial adjunct therapy in the management of heart failure, particularly for patients who have limited improvement with standard therapies.

To our knowledge, this is one of the first studies to use a prospective, double-blind, randomized controlled trial design to investigate the effects of CoQ10 supplementation on cardiac function using speckle-tracking echocardiography (STE), specifically GLS and other clinical tests like the MLHFQ and 6MWT. While other studies have focused on EF and NT-proBNP levels, the inclusion of GLS as a parameter provides a novel insight into the mechanics of left ventricular function, which is often more sensitive to early myocardial changes than traditional measures like EF. GLS is a measure of the heart’s deformation, or strain, or its ability to contract and relax in the longitudinal direction, i.e., from base to apex, and can be measured using STE (Biering-Sørensen et al., 2015) [20]. GLS detects the subtler earliest changes in cardiac function, even before the changes in EF [20,21]. Hence, it is a strong predictor of cardiovascular events, such as heart failure, myocardial infarction, chemotherapy-induced cardiotoxicity, cardiomyopathy, amyloidosis, and mortality [22,23]. It can also be useful as an efficient monitoring tool for post-therapeutic changes [24]. Furthermore, a review evaluating GLS and LAS as echocardiographic biomarkers in HFpEF patients indicates that combining these parameters can provide detailed insights into myocardial deformation, help define HFpEF phenogroups, and guide tailored therapies, suggesting that future guidelines should recommend their integrated use for comprehensive HFpEF assessments [25]. More broadly, deformation imaging techniques continue to prove their prognostic value across different cardiac diseases. In moderate-to-severe aortic stenosis, for example, Springhetti et al. showed that a peak atrial longitudinal strain (PALS) of < 16% was independently associated with death or heart-failure hospitalization—even after an adjustment for left-ventricular GLS, ejection fraction, and right-ventricular function (aHR 0.95 per 1% increase; 95% CI 0.91–0.99; *p* = 0.017). This underscores how speckle-tracking strain parameters—whether ventricular (GLS) or atrial (PALS)—provide sensitive, incremental markers of myocardial dysfunction and risk stratification beyond traditional measures [26].

## 5. Future Directions

Future research should focus on optimizing the dosage of CoQ10 supplementation to ensure the consistent plasma CoQ10 levels that lead to clinical improvements. Studies should also explore the potential of CoQ10 as an adjunct to current heart failure therapies, particularly in combination with beta-blockers, ACE inhibitors, and SGLT2 inhibitors. Larger, multicentric trials with extended follow-up periods are needed to validate these findings and assess their applicability to broader HF populations, including patients with preserved EF (HFpEF).

Additionally, the role of CoQ10 in improving endothelial function and reducing blood pressure, as noted in some studies [27,28], should be further explored, especially in patients with concomitant hypertension and HF. The potential mechanisms by which CoQ10 reduces oxidative stress and enhances nitric oxide availability in the vasculature could have broader implications for cardiovascular health.

## 6. Limitations and Areas for Further Research

Despite the promising results, this study has several limitations. The six-month follow-up period may not have been long enough to fully capture the long-term beneficial effects or adverse side-effects of CoQ10 supplementation on HF outcomes, such as mortality and hospitalization rates. Future studies with longer follow-up durations are necessary to assess the sustained benefits of CoQ10, particularly in reducing MACE and improving long-term survival. Additionally, while this study provides valuable data on Q10’s impact on cardiac function, it remains unclear which subgroups of HF patients (NYHA class, baseline CoQ10 levels) benefit the most from supplementation. Further research is needed to determine the optimal dosage and patient population for CoQ10 therapy. Finally, although NT-proBNP and other biomarker changes were measured, the small sample size and variability in GDMT compliance may limit the interpretability of these findings; larger studies are required to validate the biomarker responses to CoQ10.

## 7. Conclusions

In conclusion, this study demonstrates that CoQ10 supplementation significantly improves cardiac function and quality of life in patients with HF. These findings, combined with the existing body of literature, suggest that CoQ10 is a promising adjunct therapy for HF management. However, further research is necessary to refine the treatment protocol, particularly in terms of optimal dosage and patient selection. The promising results of this study pave the way for future investigations into the long-term benefits of CoQ10 in HF therapy.

## Figures and Tables

**Table 1:** Comparison of clinical and demographic parameters between CoQ10 and placebo groups at inclusion.

Variable	CoQ10 Group	Placebo Group	Total (N = 120)	*p*-Value
	(N = 60)	(N = 60)		
**Age**				0.624
Mean (SD)	66.5 (12.8)	67.7 (14.3)	67.1 (13.5)	
**Environment**				0.581
Rural (%)	28 (46.7%)	25 (41.7%)	53 (44.2%)	
Urban (%)	32 (53.3%)	35 (58.3%)	67 (55.8%)	
**BMI (kg/m²)**				0.56
Mean (SD)	32.4 (7.0)	33.2 (8.1)	32.8 (7.5)	
**Smoking status**				0.453
Non-smokers (%)	35 (58.3%)	39 (65.0%)	74 (61.7%)	
Smokers (%)	25 (41.7%)	21 (35.0%)	46 (38.3%)	
**Length of stay (days)**				0.625
Mean (SD)	10.7 (5.4)	10.2 (5.8)	10.4 (5.6)	
**SBP at Admission (mmHg)**				0.560
Mean (SD)	124.2 (13.3)	126.8 (18.2)	130.0 (16.9)	
**DBP at Admission (mmHg)**				0.489
Mean (SD)	78.2 (9.2)	80.3 (10.4)	77.3 (10.2)	
**HR at Admission (bpm)**				0.542
Mean (SD)	74.3 (13.1)	72.8 (13.8)	73.6 (13.4)	
**Laboratory Data**				
**NT-proBNP (pg/mL)**				0.987
Mean (SD)	1401.9 (1501.0)	1397.4 (1503.1)	1399.7 (1495.7)	
**Hemoglobin (g/dL)**				0.157
Mean (SD)	12.7 (0.7)	12.5 (1.2)	12.6 (1.0)	
**Glucose (mg/dL)**				0.101
Mean (SD)	113.0 (35.7)	126.5 (51.9)	119.7 (44.9)	
**eGFR (mL/min)**				0.114
Mean (SD)	73.1 (16.7)	69.4 (25.0)	71.25 (21.7)	
**Functional capacity**				
**GLS (%)**				0.497
Mean (SD)	−11.2 (3.8)	−11.6 (3.8)	−11.4 (3.8)	
**6MWT Distance (m)**				0.47
Mean (SD)	299.4 (102.3)	292.1 (96.8)	295.8 (100.4)	
**MLHFQ**				0.227
Mean (SD)	57.6 (16.6)	54.0 (16.1)	55.8 (16.4)	
**Comorbidities**				
**Diabetes Type 2**	25 (41.7%)	27 (45.0%)	52 (43.3%)	0.713
**Atrial Fibrillation**	33 (55.0%)	24 (40.0%)	57 (47.5%)	0.1
**COPD**	22 (36.7%)	14 (23.3%)	36 (30.0%)	0.111
**Heart Failure**	55 (91.7%)	50 (83.3%)	105 (87.5%)	0.168
**Respiratory Failure**	2 (3.3%)	6 (10.0%)	8 (6.7%)	0.143

Statistical significance was determined using a linear model ANOVA for continuous variables (BMI: Body Mass Index; SBP: Systolic Blood Pressure; DBP: Diastolic Blood Pressure; HR: Heart Rate; NT-proBNP: N-terminal pro–B-type Natriuretic Peptide) and Pearson’s Chi-squared test for categorical variables (NYHA: New York Heart Association classification; COPD: Chronic Obstructive Pulmonary Disease). Missing data is denoted by “N-Miss”. Additional parameters include the following: NT-proBNP (N-terminal pro–B-type Natriuretic Peptide), SBP (Systolic Blood Pressure), DBP (Diastolic Blood Pressure), and HR (Heart Rate). All variables are presented as Mean (SD) for continuous data and as count (%) for categorical data.

**Table 2:** Effect of comparative analysis of clinical and paraclinical parameters between baseline and endpoint for CoQ10 administration.

Variable	Baseline (N = 60)	Endpoint (N = 60)	Total (N = 120)	*p*-Value
**SBP (mmHg)**	135.8 (18.2)	124.2 (13.3)	130.0 (16.9)	<0.001
**DBP (mmHg)**	80.3 (10.4)	74.2 (9.2)	77.3 (10.2)	<0.001
**HR (bpm)**	75.3 (13.1)	71.8 (13.8)	73.5 (13.4)	0.32
**Hemoglobin (g/dL)**	12.7 (0.7)	12.5 (0.6)	12.6 (0.7)	0.026
**Blood Glucose (mg/dL)**	126.5 (51.9)	120.5 (45.3)	123.5 (48.6)	0.504
**eGFR (mL/min)**	63.4 (25.0)	73.1 (16.7)	68.2 (21.7)	0.014
**Sodium (mmol/L)**	139.9 (3.9)	140.6 (4.1)	140.2 (4.0)	0.295
**Potassium (mmol/L)**	4.1 (0.6)	4.0 (0.6)	4.0 (0.6)	0.722
**NT-proBNP (pg/mL)**	1401.9 (1501.0)	815.6 (957.4)	1108.8 (1287.7)	0.012
**LVEF (%)**	38.9 (6.9)	40.6 (6.6)	39.7 (6.8)	0.17
**GLS (%)**	−11.7 (−4.4)	−14.9 (−3.7)	−13.3 (−4.4)	<0.001
**6MWT Distance (m)**	267 (95.5)	349.3 (100.6)	277.0 (100.6)	0.008
**MLHFQ**	59.0 (14.5)	52.9 (17.1)	55.8 (16.4)	0.043
**SaO2 Initial (%)**	97.9 (1.2)	98.6 (0.6)	98.2 (1.0)	<0.001
**SaO2 Final (%)**	97.5 (1.4)	98.4 (0.7)	97.9 (1.2)	<0.001

Statistical significance was determined using a linear model ANOVA for continuous variables (SBP: Systolic Blood Pressure; DBP: Diastolic Blood Pressure; HR: Heart Rate; NT-proBNP: N-terminal pro–B-type Natriuretic Peptide; LVEF: Left Ventricular Ejection Fraction; GLS: Global Longitudinal Strain; 6MWT: 6-Minute Walk Test; SaO2: Oxygen Saturation). MLHFQ (Minnesota Living with Heart Failure Questionnaire). All variables are presented as Mean (SD) for continuous data and as count (%) for categorical data. The *p*-value threshold for significance is 0.05.

**Table 3:** Effect of comparative analysis of clinical and paraclinical parameters between baseline and endpoint for placebo administration.

Variable	Baseline (N = 60)	6 Months (N = 60)	Total (N = 120)	*p*-Value
**BMI (kg/m²)**	33.2 (8.1)	31.7 (5.7)	32.4 (7.0)	0.218
**SBP (mmHg)**	135.8 (18.2)	134.9 (17.8)	135.4 (18.0)	0.912
**DBP (mmHg)**	80.3 (10.4)	79.9 (10.2)	80.1 (10.3)	0.894
**HR (bpm)**	72.8 (13.8)	72.3 (13.5)	72.6 (13.7)	0.872
**Hemoglobin (g/dL)**	12.5 (1.2)	12.3 (1.1)	12.4 (1.1)	0.288
**Blood Glucose (mg/dL)**	126.5 (51.9)	120.5 (45.3)	123.5 (48.6)	0.504
**eGFR (ml/min)**	63.4 (25.0)	62.1 (24.2)	62.8 (24.6)	0.802
**Creatinine (mg/dL)**	1.1 (0.4)	1.2 (0.4)	1.1 (0.4)	0.845
**Sodium (mmol/L)**	140.3 (3.1)	141.0 (2.9)	140.6 (3.0)	0.224
**Potassium (mmol/L)**	4.3 (0.5)	4.4 (0.6)	4.4 (0.6)	0.42
**NT-proBNP (pg/mL)**	1401.9 (1501.0)	1378.5 (1492.4)	1390.2 (1496.7)	0.79
**LVEF (%)**	38.4 (7.3)	38.4 (7.2)	38.4 (7.2)	0.96
**6MWT (meters)**	254.7 (94.4)	267.0 (96.5)	260.8 (95.3)	0.481
**GLS (%)**	−11.2 (3.8)	−11.6 (3.8)	−11.4 (3.8)	0.497
**MLHFQ**	58.57 (14.39)	58.30 (14.68)	57.78 (14.53)	0.27
**SaO2 Initial (%)**	96.5 (4.0)	96.5 (4.1)	96.5 (4.0)	0.964
**SaO2 Final (%)**	95.2 (4.5)	95.2 (4.6)	95.2 (4.5)	0.936

Statistical significance was determined using a linear model ANOVA for continuous variables. Variables include BMI (Body Mass Index), SBP (Systolic Blood Pressure), DBP (Diastolic Blood Pressure), HR (Heart Rate), NT-proBNP (N-terminal pro–B-type Natriuretic Peptide), LVEF (Left Ventricular Ejection Fraction), GLS (Global Longitudinal Strain), 6MWT (6-Minute Walk Test), SaO2 (Oxygen Saturation), and MLHFQ (Minnesota Living with Heart Failure Questionnaire). All variables are presented as Mean (SD) for continuous data.

## Data Availability

The data are available upon request from the corresponding author under favorable conditions.
